# Plasma YKL-40 Elevation on Admission and Follow-Up Is Associated with Diastolic Dysfunction and Mortality in Patients with Acute Myocardial Infarction

**DOI:** 10.1155/2018/8701851

**Published:** 2018-03-01

**Authors:** Selcuk Pala, Munevver Sari, Gokhan Kahveci, Elnur Alizade, Ugur Arslantas, Abdulkadir Uslu

**Affiliations:** Cardiology Department, Kosuyolu Research and Education Hospital, University of Health Sciences, Denizer Street, Cevizli Intersection, Kartal, 34865 Istanbul, Turkey

## Abstract

**Objective:**

The aim of the study was to determine an association between the plasma YKL-40 level and echocardiographic left ventricle systolic and diastolic function parameters in patients with acute myocardial infarction.

**Subjects and Methods:**

The study included 46 patients with acute myocardial infarction. Serum brain natriuretic protein (BNP) and YKL-40 levels were analyzed on admission and after one month. Left ventricle systolic and diastolic functions and Tei index were computed by transthoracic echocardiography.

**Results:**

Plasma YKL-40 was significantly higher in patients with acute myocardial infarction (AMI) (101.7 *μ*g/L versus 34 *μ*g/L, resp., *p* < 0.001) and remained higher than in healthy subjects after one month. The levels of YKL-40 on admission were correlated with log BNP on admission (*r*=0.41, *p*=0.004), Tei index (*r*=0.44, *p*=0.002), left atrium volume index (*r*=0.32, *p*=0.02), and mitral septal annular E/e′ (*r*=0.44, *p*=0.003). Death was more frequently observed in patients with plasma YKL-40 above the median value than in those with plasma YKL-40 below the median value (*p*=0.001; OR = 13.6 (2.5–72.3)).

**Conclusion:**

YKL-40 elevations in patients with AMI remain at least one month and are associated with serum BNP elevations, diastolic dysfunction, and long-term increased overall mortality. It has prognostic importance in patients with AMI.

## 1. Introduction

Acute myocardial infarction (AMI) is considered to be an inflammatory disease in which the initial process involves infiltration of monocytes into the vessel wall and their subsequent differentiation into macrophages, atherosclerotic plaque rupture/erosion, and local thrombosis [1, 2]. Several biomarkers of inflammation have been investigated in the setting of AMI such as high-sensitive C-reactive protein (hs-CRP), myeloperoxidase, pregnancy-associated plasma protein A, matrix metalloproteinases, pentraxin-3, serum amyloid A, fibrinogen, cytokines, interleukins, and cell adhesion molecules [3]. The prognosis of AMI is depending on a number of clinical, echocardiographic, and biochemical markers. Thus, identification of new molecules to facilitate selection of patients at high risk for adverse clinical outcomes is important [4].

YKL-40, also known as chitinase-3-like 1 protein and human cartilage glycoprotein 39, is produced locally by macrophages inside the vessel wall, vascular smooth muscle cells, arthritic chondrocytes, inflamed tissue, and cancer cells [5]. YKL-40 is a plasma protein and has a role in inflammation, cell proliferation, fibrosis, differentiation, protection against apoptosis, angiogenesis, and tissue remodelling as a response to endothelial damage [6, 7]. Previous studies demonstrated that higher YKL-40 levels are observed in patients with myocardial infarction [8–12], stable coronary artery disease [8, 13–15], and heart failure [16]. Moreover, high serum YKL-40 levels have been found to be related with all-cause and cardiovascular mortality in patients with stable coronary artery disease [14, 17] and heart failure [18].

The present study sought to determine an association between YKL-40 and echocardiographic left ventricle systolic and diastolic function parameters in patients with AMI. Also, we paid attention whether plasma YKL-40 has an effect on prognosis in these patients.

## 2. Subjects and Methods

We included 46 patients who presented with angina or angina-like symptoms within 12 hours and were diagnosed with AMI with or without ST segment elevation in 2008. Diagnosis of ST elevation myocardial infarction (STEMI) was based on typical chest pain at rest and presence of ST segment elevations 0.1 mV in two or more contiguous leads on a standard 12-lead electrocardiogram. The non-STEMI diagnosis was relied on typical ischemic symptoms, ST segment deviation or T wave changes on electrocardiogram, detection of new segmental wall motion abnormality by imaging modality, and detection rise or fall of the serum troponin-I exceeding upper reference limit. The inclusion criteria were age more the 18 and diagnosed with AMI with or without ST elevation. The exclusion criteria were cardiogenic shock, complex congenital heart disease, serum creatinine > 2.0 mg/dl or hepatic dysfunction, known cancer, or being unwilling to join the study.

Clinical and demographic characteristics of the patients, 12-lead electrocardiogram on admission and after revascularization, hemogram, biochemistry parameters, peak troponin I results were recorded. Echocardiography was performed after primary angioplasty in the hospital and after one month according to the American Society of Echocardiography recommendations. Right and left ventricle (LV) dimensions and volumetric measurements, LV ejection fraction which was obtained by Simpson's method, and valve pathologies were examined. Early transmitral velocity (E wave) was measured by pulsed-wave Doppler. Early diastolic (e′), late diastolic (a′), and systolic (s′) velocities from the mitral septal and lateral annulus were obtained by tissue Doppler imaging (TDI). Left atrium volumes were measured using the multiple-discs method. Left atrium volume index (LAVi), septal and lateral annular E/e′ ratio (early filling/early diastolic mitral annular velocity ratio), and Tei index were computed.

Serum concentration of BNP were determined with fluorescence immunoassay method by The Alere Triage Test Product Insert, Catalog 98000XR (CLIAwaived, Inc., San Diego, California, USA). Blood samples for measurement of BNP were taken within 24 hours and after one month.

Plasma levels of YKL-40 were determined by a two-site, sandwich-type, enzyme-linked immunosorbent assay (ELISA) (Quidel, CA, USA). The sensitivity of the ELISA was 20 *μ*g/L. Blood samples for measurement of YKL-40 were collected in a EDTA tube within 24 hours after admitted to the hospital and after one month. The reference intervals for plasma YKL-40 (median 34 *μ*g/L, 5–95% 20–87) were determined in 234 healthy subjects (108 men and 126 women; median age 46; range 18–79 years) [19].

The study was approved by the regional Ethics Committee, and participants gave written informed consent.

### 2.1. Statistical Analysis

Statistical Package for the Social Sciences, version 20.0 (SPSS Inc., Chicago, IL, USA), was used for statistical analysis. All results are expressed as means ± SD or median and interquartile range (IQR), where appropriate. The qualitative variables are presented as numbers and percentage. After we divided patients into 2 groups according to the median value of baseline serum YKL-40, independent-sample Student's *t*-test has been used to compare unpaired groups with a Gaussian distribution. For the categorical variables, chi-square test was used, and if the necessary criteria were not satisfied, Fisher's exact test was used. Noncontinuous numerical variables between groups were compared through Mann–Whitney *U* test, and correlation analyses were based on Pearson and Spearman's test. Log transformed data were used for the plasma YKL-40 levels at the first month and both serum BNP levels and troponin I levels at 24 hours due to non-normal distribution. A *p* value of <0.05 was considered to be statistically significant.

## 3. Results

We have conducted our study with 46 patients, 24 (52%) of whom were diagnosed with STEMI and the remaining 22 (48%) as non-STEMI. The mean age of the patients was 58 ± 11 years. Majority of the patients were male gender (*n*=39,85%). The mean heart rate of the patients was 76 ± 18 beats/min, and blood pressure was 138 ± 27/86 ± 15 mmHg on admission. Ninety-one percent (*n*=41) of the patients were Killip class 1, and 9% (*n*=5) were Killip class 2. Five patients with STEMI received thrombolytic therapy, and for others, invasive treatment was performed. There was atrial fibrillation in 3 patients at the time of admission. The mean LV ejection fraction of the patients before discharge was 57 ± 11% and after one month was 63 ± 10%. The median mitral annular septal E/e′ on admission was 13.1 (9.4–17.2) and after one month was 11.8 (9.7–14.2).

**Figure 1:**
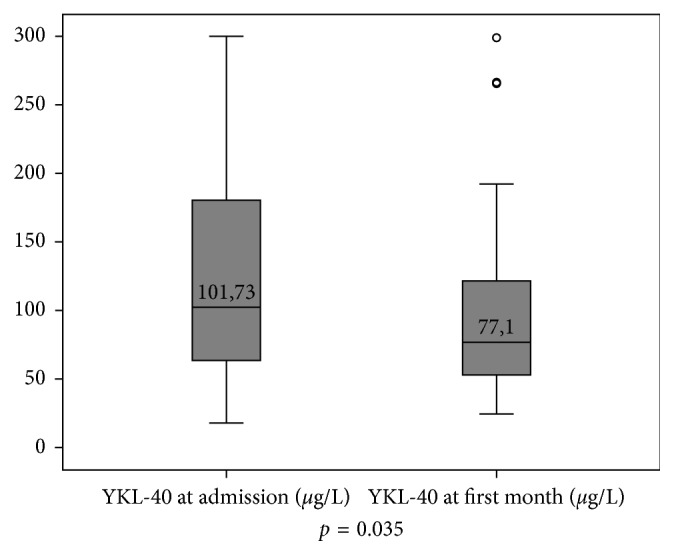
Plasma YKL-40 levels of the patients on admission and after one month. The plasma YKL-40 significantly reduced after one month (*p*=0.035).

The median YKL-40 value on admission was 101.7 (63.4–182) *μ*g/L, and the median YKL-40 value after one month was 77.1 (52.7–125.5) *μ*g/L. On admission, median plasma YKL-40 level in the patients with AMI was significantly higher compared to plasma YKL-40 in healthy subjects [19] (101.7 *μ*g/L versus 34 *μ*g/L, resp., *p* < 0.001). The level of the plasma YKL-40 significantly reduced after one month (*p*=0.035, Figure 1) but remained still higher than in healthy subjects (77.1 *μ*g/L versus 34 *μ*g/L, resp., *p* < 0.001). At the first-month follow-up, one patient died from cardiovascular etiology, 1 patient needed repeat revascularization within the first month, and 2 patients developed heart failure.

When the patients were divided into 2 groups according to median YKL-40 levels, no significant differences were observed between the groups in terms of hypertension, diabetes mellitus, hyperlipidemia, atherosclerotic vascular disease history, smoking, family history of premature coronary artery disease, be taking acetylsalicylic acid and be taking statin on admission, body mass index, and LV ejection fraction (*p* > 0.05). Also, no difference was observed in the type of myocardial infarction (STEMI or non-STEMI) between groups (*p*=0.55). Patients with plasma YKL-40 above the median value on admission had older age, higher NYHA class, higher Tei index, higher mitral septal E/e′ and they more often applied out of the office time than in those below. Moreover, same patients had higher log BNP and higher log YKL-40 at first month (Table 1).

**Figure 2:**
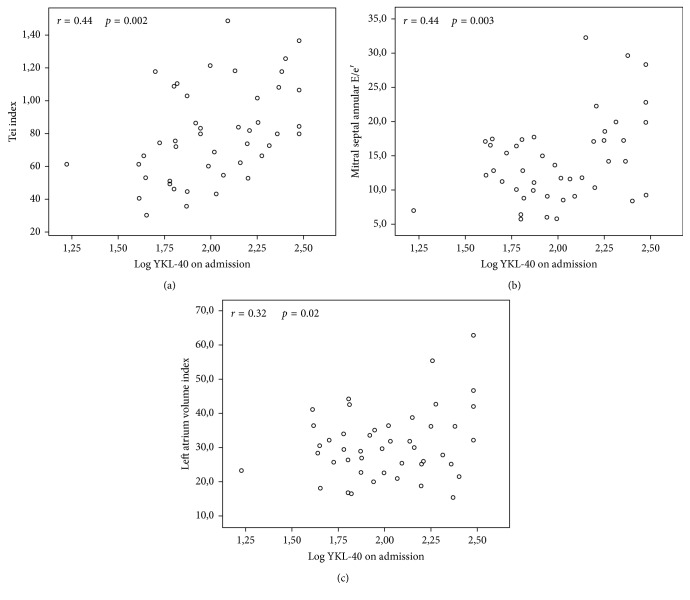
Plasma YKL-40 on admission was moderately correlated with (a) Tei index (*r*=0.44, *p*=0.002), (b) left atrium volume index (LAVi) (*r*=0.32, *p*=0.02), and (c) mitral annular septal E/e′ (*r*=0.44, *p*=0.003).

According to Pearson' correlation analysis, the level of YKL-40 on admission was at least moderately correlated with log BNP on admission (*r*=0.41, *p*=0.004), log YKL-40 at the first month (*r*=0.55, *p* < 0.001) (Figure 2), log BNP at the first month (*r*=0.42, *p*=0.006), Tei index (*r*=0.44, *p*=0.002), LAVi (*r*=0.32, *p*=0.02), and mitral septal annular E/e′ (*r*=0.44, *p*=0.003).

**Figure 3:**
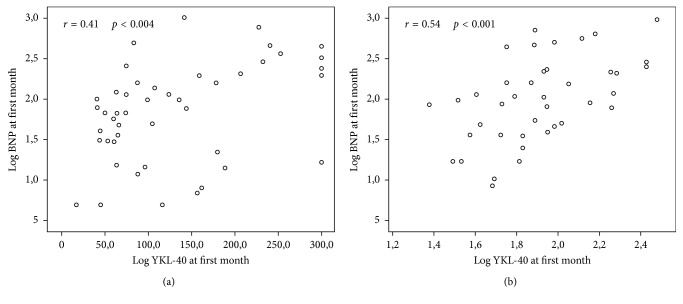
(a) Log YKL-40 on admission was moderately correlated with log BNP on admission (*r*=0.41, *p*=0.004). (b) Moreover, good correlation was observed between log YKL-40 and log BNP after one month (*r*=0.54, *p* < 0.001).

Good correlation was seen between log YKL-40 and age (*r*=0.50, *p*=0.001) and log BNP after one month (*r*=0.54, *p* < 0.001) (Figure 3). Log BNP at the first month was correlated with age, log YKL-40, LV ejection fraction, LV end-systolic volume index, LAVi, mitral septal E/e′, mitral lateral E/e′, TAPSE, right ventricle TDI systolic velocity, and right ventricle E/e′ at the first month as seen in Table 2.

After 8 years, 32% (*n*=15) of the study patients died from any cause. Overall death was more frequently observed in patients with plasma YKL-40 above the median value than in those below (*p*=0.001; OR = 13.6 (2.5–72.3)).

## 4. Discussion

In this study, we found that the plasma YKL-40 levels in patients with AMI on admission were significantly higher as compared to plasma YKL-40 in healthy participants. Furthermore, plasma YKL-40 levels reduced significantly after one month but remained still higher than in healthy subjects [19], in consistent with the previous study [11]. Copenhagen City Heart Study showed that high baseline plasma YKL-40 was significantly associated with incident-stroke, independent of hs-CRP, but not with myocardial infarction [20]. YKL-40 itself does not seem to give rise to myocardial infarction, in fact the aforementioned data could show that plasma YKL-40 increases as an acute phase response in the case of AMI in which inflammation takes part in a critical role. Unlike to CRP, which is substantially produced by hepatocytes, YKL-40 is produced by macrophages and neutrophils within inflamed tissues and thus might reflect better local vascular inflammation at the early stage of the event. Moreover, plasma YKL-40 was higher after one month as compared to healthy subjects, it could therefore be speculated that plasma YKL-40 may play a role in the regenerative process in both the acute and subacute phase. Previously, it was made clear that CRP and YKL-40 positively correlated in the early stage of AMI but not at the first month [10, 11], suggesting that noninflammatory properties of YKL-40 such as cell proliferation, fibrosis, differentiation, protection against apoptosis, angiogenesis, and tissue remodelling may have a role after acute phase.

The lack of relation between plasma YKL-40 levels and troponin I level in 24 hours suggests that cardiomyocytes are not major source of YKL-40. Wang et al. described that YKL-40 gene expression was similar in ischemic and nonischemic myocardium [11] and Hedegaard et al. concluded that maximum plasma YKL-40 was not associated with infarct size, assessed by magnetic resonance imaging in patients with STEMI [10].

It is known that plasma YKL-40 is correlated well with ageing as we found and serum YKL- 40 is a prognostic biomarker for all-cause mortality in octogenarians [21]. We sorted out that patients with higher admission YKL-40 were more symptomatic had higher Tei index, which indicates globally LV systolic and diastolic function and had higher mitral septal E/e′, that gives information about LV filling pressure. Also, these patients had a trend toward high log BNP at admission but significantly higher log BNP after one month, this supports preceding findings. BNP have primarily been dedicated to the diagnosis, prognosis, and monitoring treatment of congestive heart failure and have also been pointed out to be increased in the setting of AMI [22–24]. BNP is released from heart in response to increased ventricular wall stress [25]. Elevated BNP after AMI identifies patients at risk of adverse left ventricular remodelling, chronic left ventricular dysfunction, and congestive heart failure [25–27]. It is well known that BNP levels are correlated with age, renal function, intracardiac pressures, and ejection fraction [25].

We found that plasma YKL-40 levels on admission were moderately correlated with log YKL-40 at first month; log BNP on admission and at first month; Tei index, LAVi, and mitral septal E/e′ during hospitalization; and LAVi, mitral septal E/e′, and mitral lateral annular E/e′ at first month. There seemed an association between baseline YKL-40 and diastolic function parameters but not LV ejection fraction. Interestingly, Nøjgaard et al. made mention of no correlation between serum YKL-40 and ejection fraction and other echocardiographic indices of systolic or diastolic heart function during follow-up in patients with AMI [12]. Log BNP after one month was associated with both systolic and diastolic functions in accordance with previous studies [25, 26] and log YKL-40 at the first month. Hedegaard et al. also concluded plasma YKL-40 may be an indirect marker of LV ejection fraction recovery, independent of hs-CRP, and higher plasma YKL-40 was related with lower recovery. YKL-40 does not seem to have a direct effect on LV ejection fraction [10].

To our knowledge, this is the first report demonstrating a relationship plasma YKL-40 and diastolic dysfunction. Chen et al. showed that left ventricle diastolic dysfunction was observed in most of AMI patients even after successful invasive treatment [26]. Yoon et al. described that age, increased NT-proBNP, and impaired diastolic recovery were independent predictors of major adverse cardiovascular events in 463 patients with preserved LV systolic function at 6 months after AMI [25]. Although they found an improvement in LV systolic function, LV diastolic function had not improved in 47.9% of the patients by the 6-month follow-up after AMI. AMI results in acute derangement of myocardial contraction and relaxation mechanics induced by regional wall motion abnormality, interstitial edema, fibro-cellular infiltration, ischemia, and scar formation. It was shown that myocardial stunning involves changes in both systolic and diastolic component. LV diastolic dysfunction is an earlier, more sensitive sign of myocardial ischemia and persists longer. Diastolic function should be evaluated with PW Doppler velocities, time intervals, TDI parameters, and LA volume. LA volume is less influenced by acute hemodynamic changes, so it is plausible to incorporate LAVi when interpreting diastolic function in patients with AMI. An elevated E/e′ ratio and increased LAVi can estimate increased LV filling pressures [27]. The present study could point out that YKL-40 were associated with diastolic dysfunction especially at early stage (Tei index, LAVi, and mitral septal E/e′). Higher YKL-40 may represent the more intense local inflammation at the tissue level or may be a compensatory mechanism.

All-cause mortality was observed more frequently in patients with the level of plasma YKL-40 above the median value than those below (*p*=0.001; OR = 13.6 (2.5–72.3)) (Table 1). High plasma YKL-40 on admission may be speculated as a prognostic marker in the AMI setting taking into consideration previous studies in patients with AMI [9] and in patients with stable coronary artery disease [14].

The most important limitation of this study is the small number of patients. Eight-years follow-up mortality due to any causes was obtained from the public death reporting system, and exact cause of the mortality was not achieved. Regression analysis was not performed because the number of the study patients was low. Reevaluation of systolic and diastolic functions at the 6th month after AMI could give more clear information. It should be supported by larger scale studies to increase the clinical use of these data.

## 5. Conclusion

Plasma YKL-40 was significantly higher in patients with AMI and remained still higher at the first month than healthy subjects. There was a significant correlation between high plasma YKL-40 on admission and diastolic dysfunction. Overall mortality was observed more frequently in patients with the YKL-40 level above the median value. YKL-40 may provide prognostic information with regard to diastolic function and overall mortality in patients with AMI.

## Figures and Tables

**Table 1:** Demographic characteristics of the patients which were divided into 2 groups by median YKL-40 level on admission.

	Patients with plasma YKL-40 below the median value	Patients with plasma YKL-40 above the median value	*p*
Age, years	54 ± 9	63 ± 11	0.01
Gender, male, *n* (%)	21/23 (91%)	18/23 (78%)	0.21
Hypertension, *n*/total (%)	10/23 (43%)	8/23 (35%)	0.54
Diabetes mellitus, *n*/total (%)	5/23 (22%)	7/23 (30%)	0.50
CVH history, *n*/total (%)	8/23 (34%)	10/23 (43%)	0.24
Hyperlipidemia, *n*/total (%)	10/23 (43%)	4/23 (17%)	0.05
Body mass index, kg/m^2^	28.9 ± 4.8	27.7 ± 3.6	0.35
AF at admission, *n*/total (%)	0/23	3/23 (13%)	0.07
STEMI, *n* (%) Non-STEMI, *n* (%)	11 (48%) 12 (52%)	13 (56%) 10 (44%)	0.55
Application within office time, *n* Application out of office time, *n*	14 (60%) 9 (40%)	6 (26%) 17 (74%)	0.01
NYHA class I, *n*/total (%) NYHA class II-III, *n*/total (%)	21 (91%) 2 (9%)	12 (52%) 11 (48%)	0.003
Systolic blood pressure	142 ± 32	134 ± 21	0.38
Serum glucose, mg/dl	113 (92–167)	152 (114–246)	0.06
Serum creatinine, mg/dl	1.05 ± 0.2	1.2 ± 0.3	0.06
White blood cell counts	11.09 ± 3.6	10.11 ± 3.7	0.39
Hemogram, mg/dl	14.5 ± 1.6	13 ± 1.7	0.09
Log troponin-I 24 hours	1.4 ± 0.5	1.5 ± 0.8	0.71
Log BNP on admission	1.6 ± 0.4	2.0 ± 0.6	0.06
First-month log BNP	1.8 ± 0.5	2.1 ± 0.4	0.04
First-month log YKL-40	1.7 ± 0.2	2.0 ± 0.2	0.004
EF (Simpson's method), %	58 ± 10	57 ± 12	0.31
Tei index	0.71 ± 0.26	0.88 ± 0.28	0.03
LAVi, ml/m^2^	28.9 ± 7.8	33.1 ± 11.6	0.15
Mitral lateral e′, cm/s	9.3 ± 3.3	7.7 ± 3.3	0.11
Lateral E/e′, median (IQR)	9.6 (6.4–12.3)	9.6 (8.5–18.4)	0.14
Mitral septal e′, cm/s	7.3 ± 2.5	5.7 ± 2.0	0.02
Septal E/e′	11.9 ± 4.1	16.8 ± 7.1	0.007
RV TDI s′, cm/s	14.6 ± 4.3	12.6 ± 3.4	0.08
TAPSE, cm	2.2 ± 0.4	2.3 ± 0.6	0.50
All-cause death	2/23 (9%)	12/23 (52%)	0.001

ASA: acetylsalicylic acid; AF: atrial fibrillation; BNP: brain natriuretic peptide; CVH: cardiovascular hastalık; EF: ejection fraction; LAVi: left atrium volume index; NYHA: New York Heart Association; RV: right ventricle; STEMI: ST elevation myocardial infarction.

**Table 2:** Results of correlation analyses in patients with acute myocardial infarction.

	YKL-40 on admission	Log YKL-40 at the first month	Log BNP at the first month
Age	*r*=0.49, *p* < 0.001	*r*=0.50, *p*=0.001	*r*=0.64, *p* < 0.001
Log YKL-40 at admission		*r*=0.55, *p* < 0.001	
Log YKL-40 at 1 month	*r*=0.55, *p* < 0.001		*r*=0.54, *p* < 0.001
Log BNP at admission	*r*=0.41, *p*=0.004	*r*=0.39, *p*=0.01	
Log BNP at 1 month	*r*=0.42, *p*=0.006	*r*=0.54, *p* < 0.001	
LAVi	*r*=0.32, *p*=0.02		
Tei index	*r*=0.44, *p*=0.002		
Mitral lateral s′	*r*=−0.30, *p*=0.04		
Mitral lateral e′	*r*=−0.30, *p*=0.04		
Mitral septal s′	*r*=−0.40, *p*=0.006		
Mitral septal e′	*r*=−0.42, *p*=0.004		
Mitral septal e/e′	*r*=0.44, *p*=0.003		
LAVi at 1 month	*r*=0.33, *p*=0.03		*r*=0.44, *p*=0.004
Ejection fraction at 1 month			*r*=−0.45, *p*=0.004
LVESVi at 1 month			*r*=0.41, *p*=0.00
Mitral lateral s′ at 1 month		*r*=−0.36, *p*=0.02	*r*=−0.52, *p*=0.001
Mitral lateral e′ at 1 month	*r*=−0.40, *p*=0.01	*r*=−0.39, *p*=0.01	*r*=−0.49, *p*=0.001
Lateral e/e′ at 1 month	*r*=0.33, *p*=0.03		*r*=0.43, *p*=0.005
Mitral septal s′ at 1 month		*r*=−0.34, *p*=0.03	*r*=−0.47, *p*=0.002
Mitral septal e′ at 1 month	*r*=−0.47, *p*=0.002	*r*=−0.43, *p*=0.005	*r*=−0.54, *p* < 0.001
Septal e/e′ at 1 month	*r*=0.35, *p*=0.02		*r*=0.40, *p*=0.01
RV TDI s′ at 1 month			*r*=−0.38, *p*=0.01
TAPSE at 1 month			*r*=0.49, *p*=0.002
RV e/e′ at 1 month		*r*=0.37, *p*=0.02	*r*=0.33, *p*=0.03

LAVi: left atrium volume index; LVESVi: left ventricle end-systolic volume index; RV: right ventricle; RV TDI: right ventricle tissue Doppler imaging, TAPSE: tricuspid annular plane systolic excursion.
